# Association Between Neddylation and Immune Response

**DOI:** 10.3389/fcell.2022.890121

**Published:** 2022-05-05

**Authors:** Jiali Zhu, Feng Chu, Meirong Zhang, Wenhuan Sun, Fangfang Zhou

**Affiliations:** Institutes of Biology and Medical Science, Soochow University, Suzhou, China

**Keywords:** neddylation, NEDD8, immune response, innate immune cells, anti-viral pathway

## Abstract

Neddylation is a ubiquitin-like post-translational protein modification. It occurs *via* the activation of the neural precursor cell expressed, developmentally downregulated protein 8 (NEDD8) by three enzymes: activating enzyme, conjugating enzyme, and ligase. NEDD8 was first isolated from the mouse brain in 1992 and was initially considered important for the development and differentiation of the central nervous system. Previously, the downregulation of neddylation was associated with some human diseases, such as neurodegenerative disorders and cancers. In recent years, neddylation has also been proven to be pivotal in various processes of the human immune system, including the regulation of inflammation, bacterial infection, viral infection, and T cell function. Additionally, NEDD8 was found to act on proteins that can affect viral transcription, leading to impaired infectivity. Here, we focused on the influence of neddylation on the innate and adaptive immune responses.

## Introduction

**FIGURE 1:**
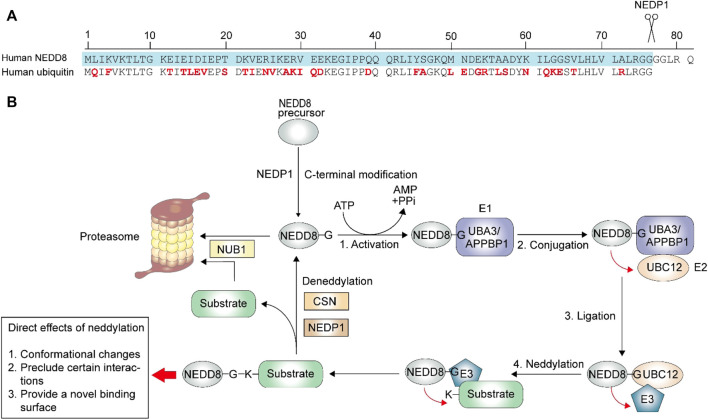
Neddylation process. The neddylation process is similar to ubiquitination, with a three-step enzymatic reaction. **(A)** The maturation of the NEDD8 is a C-terminal hydrolytic activity by NEDP1. The ubiquitin contains 76 amino acids, and the different residues between matured NEDD8 (blue) and ubiquitin is highlighted in red. **(B)** After maturation, NEDD8 binds to the activating enzyme E1 in an ATP-dependent manner. NEDD8 is then accepted by conjugating enzyme E2. Finally, E2 transfers NEDD8 to ligase E3, which then links the glycine of NEDD8 and the lysine residues of its substrates *via* an isopeptide bond. The following deneddylation process deconjugates NEDD8 from the neddylated substrate. And after deconjugation, NUB1 (Negative regulator of ubiquitin like proteins 1) directs NEDD8 and substrate (like CRLs) to proteasome for degradation. The direct effects of neddylation on its substrates include three parts: conformational changes, preclude certain interactions and provide a novel binding surface.

Neddylation is a form of post-translational protein modifications (PTMs) in which the ubiquitin-like protein neural precursor cell expressed, developmentally downregulated protein 8 (NEDD8) binds to the target protein *via* a process similar to ubiquitination (Yu et al., 2019; Zhao et al., 2021). It was reported that the sequences of NEDD8 and ubiquitin are 59% identical (Figure 1A) (Enchev et al., 2015). NEDD8 was first isolated from the mouse brain, (Kumar et al., 1992). The first identified substrate was Cdc53, which is a yeast cullin (Gao et al., 2006). To date, the best-studied neddylation substrates are those from the largest ubiquitin E3 ligase family called cullin-RING ligases (CRLs) (Enchev et al., 2015), which are activated by conformational changes at the C-terminal (Duda et al., 2008; Mohanty et al., 2021). NEDD8 has also been found to act on other substrates, known as non-cullin proteins, to impact gene expression, cell survival, organ development, and stress response (Enchev et al., 2015). Once CRLs are activated, various cellular substrates participate in innate immune responses, cell cycle regulation, and cytoskeleton modeling (Mohanty et al., 2021).

In 1998, Cullin3 (Cul3) and Cul-4A were observed to be highly expressed in cultured colon cancer cells and primary breast cancer (Chen et al., 1998; Du et al., 1998). Elevated expression of NEDD8 was then observed in various human tumor cell lines, including leukemia cells and HeLa (Hori et al., 1999). Both these findings confirmed that neddylation is relevant to cancer progression. Some neddylated non-cullin proteins, such as the neddylated tumor suppressor phosphatase and tensin homolog (PTEN) and breast cancer-associated protein 3 (BCA3) have been shown to be cancer promoters (Mo et al., 2016; Xie et al., 2021). In 2009, it was found that inhibition of the activating enzyme (E1) of neddylation using MLN4924 (Pevonedistat) can suppress tumor progression, and it is currently undergoing clinical trials, in combination with chemotherapies, against various types of malignant tumors (Soucy et al., 2009; Xie et al., 2021) (NCT04090736, NCT03745352). There is a large body of evidence demonstrating that MLN4924 functions as a tumor inhibitor by triggering DNA-damage responses, cell cycle arrest, apoptosis, autophagy, and alteration of mitochondrial function (Soucy et al., 2009; Zhao et al., 2012; Zhou et al., 2019).

During the past decade, the connection between neddylation and immunity has been investigated, describing the importance of this type of PTM in controlling immune responses and immune-related diseases. In this review, we summarize the neddylation process and its regulatory effects on innate and adaptive immunity.

## Neddylation Process

The primary product of the *NEDD8* gene is a NEDD8 precursor, which needs to be modified to expose the C-terminal glycine before it acts on its targets (Enchev et al., 2015). NEDD8 precursor contains 81 amino acids (Figure 1A), and NEDD8 protease 1 (NEDP1, also known as the human deneddylase 1, DEN1), is involved in NEDD8 precursor processing (Figure 1B). After the proteolytic process, the NEDD8 activating enzyme (NAE, E1) facilitates NEDD8 activation in an ATP-dependent manner, and a high-energy intermediate is produced. Following this, the conjugating enzyme (E2) accepts and transfers NEDD8 to ligase (E3), which ensures specific conjugation between activated NEDD8 and its target protein (Wu et al., 2003; Rabut and Peter, 2008). Similar to ubiquitin, the final attachment occurs *via* an isopeptide linkage between conserved C-terminal glycine 76 of NEDD8 and the lysine residue of its substrates (Rabut and Peter, 2008; Watson et al., 2011). Previous studies have identified APPBP1-UBA3 (amyloid β precursor protein binding protein 1-ubiquitin activating enzyme 3) complex as E1, UBC12/UBE2M (ubiquitin conjugating enzyme E2 M) and UBE2F as E2, RING-box protein 1 (RBX1)/ regulator of cullins 1 (ROC1), RBX2/ROC2, and murine double minute 2 (MDM2) as E3 (Rabut and Peter, 2008; Li et al., 2019). Interestingly, some enzymes involved in neddylation also participate in the ubiquitination process, like MDM2 (Brooks and Gu, 2006), and the ubiquitin RING-class E3 component is not only the target of NEDD8 but also serves as E3 in the neddylation pathway (Huang et al., 2004). The following deneddylation is to separate the conjugated NEDD8 and substrate, during which the COP9 signalosome (CSN) and NEDP1 serve as deneddylase (Rabut and Peter, 2008).

## Neddylation and Innate Immune Cells

**FIGURE 2:**
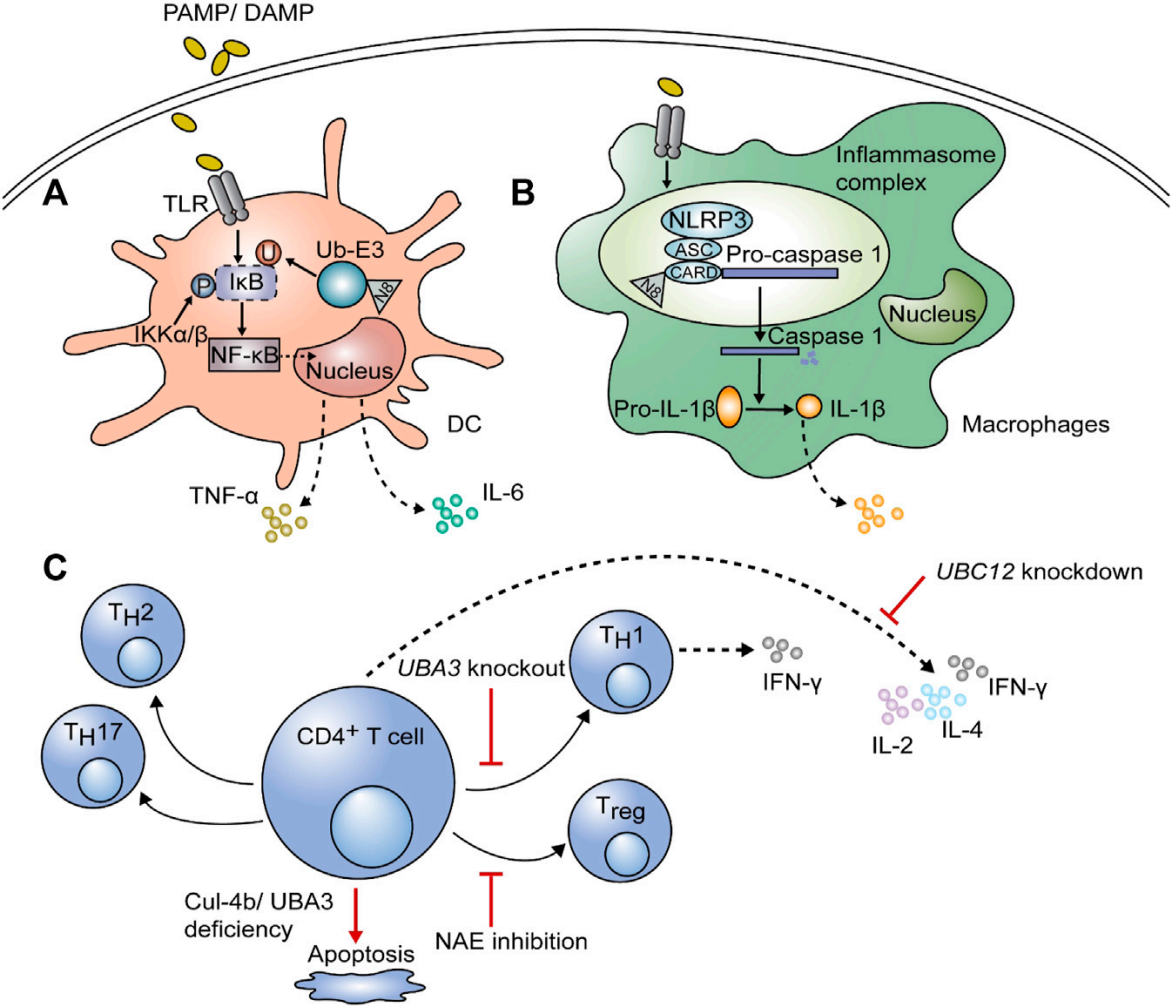
The function of neddylation on immune cells. After stimulation with pathogen-associated molecular patterns or damage-associated molecular patterns (PAMPs or DAMPs), neddylation is required to induce proinflammatory cytokines, including interleukin-6 (IL-6) and tumor necrosis factor-α (TNF-α), in dendritic cells (DCs) and macrophages. **(A)** After ubiquitination and degradation of inhibitor κB (IκB), nuclear factor (NF)-κB translocates into the nucleus and initiates gene expression; this process is dependent on the neddylation of ubiquitin E3 ligase. **(B)** The NLR family pyrin domain containing 3 (NLRP3) in the inflammasome complex of macrophages can be neddylated at the caspase recruitment domain (CARD), leading to self-cleavage of pro-caspase-1 and maturation of IL-1β, thus affecting inflammation. **(C)** For adaptive immune cells, inhibition of neddylation by knockdown of *UBC12* blocks cytokine secretion by CD4^+^ T cells, including that of IL-2, IL-4, and IFN-γ. Knockout of *UBA3* can downregulate interferon (IFN)-γ-producing T_H_1 cells and even result in apoptosis. NEDD8-activating enzyme (NAE) inhibition can regulate the polarization of CD4^+^ T cells with lower T_reg_ differentiation and a shift towards the T_H_1 phenotype. The deficiency of Cul-4b also has negative effects on CD4^+^ T cell survival.

Dendritic cells (DCs) are the most potent antigen-presenting cells (APCs) involved in the innate immunity. Once they are stimulated by pathogens, the inhibitor κB (IκB) is phosphorylated by IκB kinase (IKKβ or α), and then ubiquitinated and degraded by the E3 ligase complex. CRL-1 is a component of the E3 ligase complex, which consists of Cul1, RING box protein (SAG), and S-phase kinase-associated protein 1 (SKP1) (Emanuele et al., 2011). Ubiquitination and degradation of IκB are dependent on the neddylation of Cul1 at the C-terminal lysine residue *via* conjugation to activated NEDD8 (Figure 2A) (Xirodimas, 2008). Upon degradation of IκB, nuclear factor-κB (NF-κB) is activated; it translocates into the nucleus, leading to an increase in the transcription and secretion of proinflammatory cytokines, including interleukin-6 (IL-6) and tumor necrosis factor-α (TNF-α) (Mathewson et al., 2013; Jiang et al., 2021). Besides, neddylation is of great significance for survival of DCs. On the one hand, it was confirmed that knockdown of the key genes in neddylation pathway (*Cullin-1*, *Cullin-5*, *SENP8*, and *NEDD8*) can promote apoptosis and autophagy in *Mycobacterium tuberculosis* antigen stimulated DCs (Singhal et al., 2012; Chadha et al., 2015). On the other hand, Mathewson et al. found that inhibition of neddylation by MLN4924 treatment for 24 h or knockdown of *SAG* impairs the function of DCs without affecting the MAPK/ERK (Mitogen-activated protein kinase/Extracellular signal-related kinase) pathway and cell viability (Mathewson et al., 2013). However, long term MLN4924 admission was proven to reduce the number of APCs significantly, including DCs and macrophages, whereas showed minimal effect on T cells and B cells (Pai et al., 2017). Therefore, the effects of MLN4924 on survival of DCs is time-dependent. Importantly, MLN4924 was noticed to sensitize apoptosis and necroptosis of monocytes and immature DCs (iDCs) induced by TNF, and this effect is closely correlates with the suppressed expression of A20 (a ubiquitination editing enzyme), cellular inhibitor of apoptosis protein 2 (cIAP2), TNF receptor associated factor 1 (TRAF1) and FLIP (FLICE inhibitory protein), which provide cells resistance to TNF-induced cell death (El-Mesery et al., 2015).

Same as in DCs, lipopolysaccharide (LPS)-induced proinflammatory cytokine production can be suppressed by MLN4924, inhibiting ubiquitination and degradation of IκBα, thus impairing nuclear translocation of NF-κB (Chang et al., 2012). Besides, the NOD-like receptor family pyrin domain containing 3 (NLRP3)/apoptosis-associated speck-like protein (ASC) is associated with pro-caspase-1 *via* the caspase recruitment domain (CARD) in inflammasome complex. The activity of NEDD8 on CARD is necessary for pro-caspase-1 to self-cleave into caspase-1 (Figure 2B), which is followed by the maturation of pro-IL -1β to IL-1β (Segovia et al., 2015; Zhou et al., 2019; Swanson et al., 2019; Jiang et al., 2021). Watahiki et al. also found that MLN4924 treatment prevents LPS-stimulated *Il1b* gene expression, thus could be a new strategy for inflammatory diseases (Watahiki et al., 2020). Except for affecting inflammatory responses, MLN4924 also promotes polarization towards M2 macrophages (Asare et al., 2017). The neddylation pathway was found to be activated during methicillin-resistant *Staphylococcus aureus* (MRSA) infection, providing protection through the NEDD8-Cullin3-Nrf2-ROS axis and increased reactive oxygen species (ROS) in mouse peritoneal macrophages (Xiu et al., 2021). Moreover, neddylation inhibition was proved to decrease macrophage tumor infiltration through chemotactic cytokine ligand 2 (CCL2) reduction, thus modulates the tumor microenvironment and could be a potent cancer therapy (Zhou et al., 2019; Meerang et al., 2020). Besides, CCL5 accumulation due to MLN4924 led to M2 macrophage infiltration, and exacerbates chronic pancreatitis (Lin et al., 2021). Apart from functional effects, neddylation also matters in survival of macrophages. It was reported that partial treatment using MLN4924 diminishes TNF-α and IL-6 induced by LPS without impairing cell viability, while persistent treatment inhibited cell proliferation because of G2 cell-cycle arrest and apoptosis in RAW264.7 macrophages (Li et al., 2013). This phenomenon is mainly due to blockade of cullin neddylation, leading to inactivation of CRL E3 ligase, accumulation of cell-cycle inhibitory CRL substrates (including Wee1, p21, and p27) and induction of DNA damage (Li et al., 2013). Cycle-inhibiting factor homolog in *Burkholderia pseudomallei* (CHBP) is a bacterial deamidase effector, which recognizes the host NEDD8 and catalyzes its deamidation and triggers macrophage-specific apoptosis but preserves integrity of cell membrane (Yao et al., 2012). Hence, CHBP may has similar effect like MLN4924 which needs further research.

Neutrophils, a type of polymorphonuclear leukocytes, are myeloid lineage cells that are recruited to specific sites as the first line of innate immune responses against pathogens (Kolaczkowska and Kubes, 2013). It has been reported that neutrophil and monocyte counts in the blood increased because of MLN4924 treatment (Asare et al., 2017). Earlier study claimed that MLN4924 treatment inhibits neutrophil function of TNF-α, IL-6, and IL-1β production in a dose-dependent manner by suppressing NF-κB signaling pathway (Jin et al., 2018). Xiong et al. found that SAG deficiency dramatically increases the levels of TNF-α, but did not influence the translocation of NF-κB in neutrophils, differing from the effects observed in macrophages (Xiong et al., 2018). Since SAG is one of the targets of NEDD8 it is worth investigating whether or how neddylation acts on neutrophils.

These results suggest that neddylation can regulate the secretion of proinflammatory cytokines and proliferation in innate immune cells, as well as other aspects, such as migration and polarization of macrophages. Since adequate evidence proved that neddylation inhibition by either gene (*Cullin-1*, *Cullin-5*, *SENP8*, and *NEDD8*) knockdown or MLN4924 can result in death of innate immune cells, when MLN4924 is involved in the therapeutic treatment, like cancer treatment, the patient’s immune system needs to be closely monitored.

## Neddylation and Anti-Viral Pathways

**FIGURE 3:**
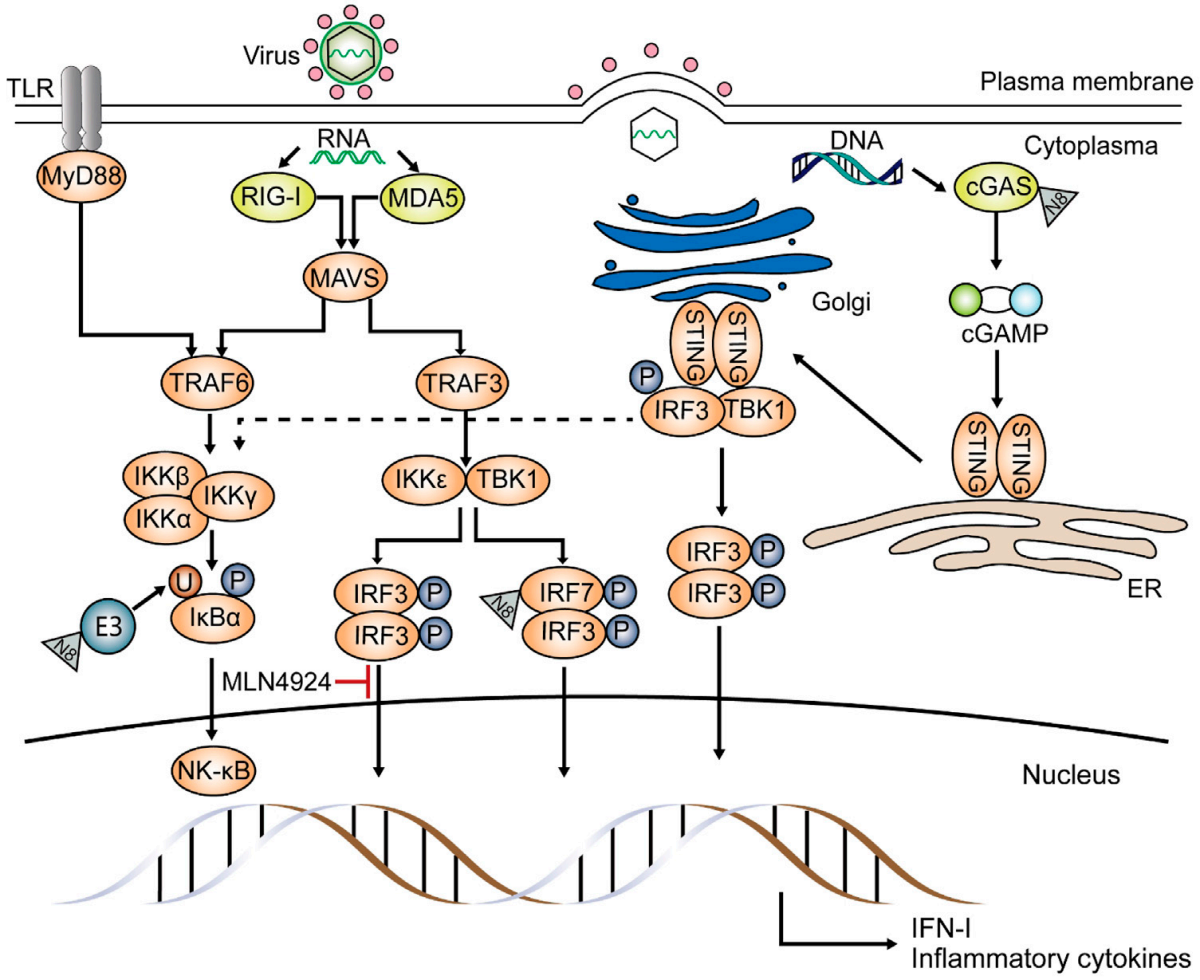
Effects of neddylation on the anti-viral pathway. Neddylation is pivotal in the antiviral pathway, inducing inflammatory cytokines and type I-IFN. Upon infection by RNA viruses, such as Sendai virus (SeV), NEDD8 either acts on ubiquitin E3 ligase and affects NF-κB translocation or binds to IRF7 directly and promotes inflammatory cytokine and type I IFN production. Moreover, neddylation is involved in the conjugation of interferon regulatory factor 3 (IRF3) and the IFN-β promoter, and this was proved using MLN4924. If invaders are DNA viruses, such as herpes simplex virus, NEDD8 targets the DNA sensor cyclic GMP-AMP synthase (cGAS) and converts it to cGAMP. cGAMP then attaches to the stimulator of interferon genes (STING) and activates TANK-binding kinase 1 (TBK1) and IRF3, activating the type I-IFN gene. STING signaling also results in the expression of inflammatory cytokines through the NF-κB pathway (dashed line). Other proteins involved in the innate antiviral pathway have not yet been reported as substrates of neddylation.

Zhao et al. used mouse models with myeloid deficiency in UBA3 or NEDD8 to study the effects of neddylation on the response against RNA virus, and they found UBA3 absence results in impaired IFN-β (interferon-β) as well as IFN-α production in myeloid dendritic cells (mDCs), proposing that myeloid neddylation is required to induce IFN production upon Sendai virus (SeV) infection (Zhao et al., 2021). Previous studies on zebrafish demonstrated that both interferon regulatory factor 3 (IRF3) and IRF7 are potential substrates of neddylation during spring viremia of carp virus (SVCV) infection and can activate anti-viral responses (Yu et al., 2019). They also claimed that neddylation inhibition increases zebrafish sensitivity to SVCV infection. And tests about SeV infection showed similar results, demonstrating that during infection, NEDD8 directly targets the C-terminal lysine residues of IRF7 (Figure 3) and partially improves its transcription by inhibiting its dimerization with the IFN-α repressor, IRF5 (Zhao et al., 2021). Through neddylation, induction of type I IFN by RNA virus is promoted, especially that of IFN-α. And research about MLN4924 demonstrated that neddylation is necessary for IRF3 to bind to the IFN-β promoter during SeV infection in HEK-293T, but the exact mechanism remains unclear (Song et al., 2016). Different from that, another study on SeV claimed that degradation of IRF3 is due to C-terminal phosphorylation by polyubiquitinated TANK-binding kinase 1 (TBK1), which is induced by a neddylated cullin-based ubiquitin ligase (Bibeau-Poirier et al., 2006). Although neddylation of IRF3 and IRF7 has been confirmed in different species, its function in the key factors in the innate immune pathway, such as melanoma differentiation-associated protein 5 (MDA5), mitochondrial antiviral-signaling protein (MAVS), and TBK1 has not yet been understood (Yu et al., 2019).

The DNA sensor, cyclic GMP-AMP synthase (cGAS), plays a fundamental role in viral DNA recognition. After cGAS is activated by combining with cytosolic DNA, ATP, and GTP and then converted into the cyclic GMP-AMP (cGAMP), the stimulator of interferon genes (STING) is activated, then triggers the following immune responses (Li et al., 2013; Wang et al., 2014; Hopfner and Hornung, 2020). The NEDD8 E3 ligase Rnf111 (Arkadia, or ring finger 111) has been shown to neddylate cGAS at numerous lysine sites upon herpes simplex virus 1 (HSV-1) infection (Figure 3), promoting its dimerization and DNA-binding capacity (Li et al., 2021). This research also proposed that neddylation inhibition by MLN4924 or deficiency of UBE2M or Rnf111 can weaken the stimulation of cGAS-STING. Another study demonstrated that MLN4924 treatment impairs HSV-1-induced NF-κB activation; this phenomenon can only be detected in the early phase of infection without affecting the activation of IRF3 and becomes inefficient in the later phase (Zhang et al., 2016).

Aside from acting directly on the anti-viral pathway, studies have demonstrated that the life cycle of viruses, including human immunodeficiency virus (HIV), Influenza A virus (IAV), and Hepatitis B virus (HBV), can be regulated by neddylation, mostly by targeting the replication process (Dias et al., 2009; Stanley et al., 2012; Liu et al., 2017). For example, the viral infectivity factor (Vif) of HIV needs UBE2F, the neddylation E2, to counteract the cytidine deaminases A3G. Hence, neddylation inhibition, using MLN4924 or knockdown of UBE2F, can suppress HIV replication (Stanley et al., 2012). HIV-2 viral protein X (Vpx) mediates depletion of the restriction factor SAM domain and HD domain-containing protein 1 (SAMHD1) *via* CRL4 (DCAF1) E3 ligase, and impaired neddylation can block this pathway, thus interfered HIV infection (Nekorchuk et al., 2013; Wei et al., 2014; Wang et al., 2017). The M1 protein and polymerase basic protein 2 (PB2, the component of RNA-dependent RNA polymerase) of IAV can be neddylated, causing reduced stability and inhibition of IAV replication (Dias et al., 2009; Zhang et al., 2017; Li et al., 2020). As for HBV, a double-stranded DNA virus, it was showed that neddylation of HBV regulatory X protein (HBx) at residues K91 and K95 by MDM2 can improve its stability and chromatin localization, thereby favoring viral replication (Liu et al., 2017). Recent research also found that HBV replication can be suppressed by NEDD8 knockdown and MLN4924 admission (Abounouh et al., 2022). In addition, MLN4924 treatment can restrain both its replication and antigen production. This is mediated by the activation of the ERK to inhibit necessary transcriptional factors, including hepatocyte nuclear factor 1 α (HNF1α), CCAAT/enhancer-binding protein (C/EBPα), and HNF4α (Xie et al., 2021). Furthermore, neddylation was noted to be required for other viruses such as Human enteroviruses and Kaposi’s sarcoma-associated herpesvirus (KSHV), making MLN4924 an promising anti-viral treatment (Hughes et al., 2015; Chang et al., 2017; Zhang et al., 2021).

There is ample evidence showing that neddylation is sometimes required to fight against viral invasion, but there are conflicting results. One report claimed that NEDD8 knockdown does not affect LPS- or SeV-induced IFN-β production in HeLa, HEK-293T, and THP-1 cells (Song et al., 2016), differing from the results in mDC mentioned above. And considering that pretreatment of HEK-293T with MLN4924 can inhibit IRF3 bind to IFN-β promoter during SeV infection, it is reasonable to assume that NEDD8 deficiency affects IFN-β production could be different in different cell lines, and MLN4924 suppresses IFN-β production in a neddylation-independent manner, but both assumptions need more evidence. Therefore, whether neddylation can benefit type I IFN production when facing virus infection and whether neddylation blockade by MLN4924 is the ideal therapeutic method for virus infection should be researched further.

## Neddylation and Adaptive Immunity

Neddylation was also shown to regulate adaptive immunity as well as innate immunity (Figure 2C). Knockdown of *UBC12* in CD4^+^ T cells results in impaired cell proliferation; suppressed production of cytokines, including IL-2, IL-4, and IFN-γ; and activation of ERK (Jin et al., 2013). Decreased IFN-γ-producing T_H_1 cells were also detected in *UBA3* knockout mice, causing less resistance to early phase parasitic infection by *Plasmodium yoelii* 17XNL (Cheng et al., 2018). They also proved that neddylation is necessary for T cell survival by suppressing mitochondria-dependent apoptosis induced by B-cell lymphoma-2 (Bcl-2). T-cell-specific, *SAG* genetic knockout animal shows normal mature T cell development, but their T cells show significantly declined activation, proliferation and T-effector cytokine release. And MLN4924 treatment showed similar *in vitro* and *in vivo* results (Mathewson et al., 2016). MLN4924 inhibits the NEDD8-activating enzyme, which then regulate T cell polarization in chronic lymphocytic leukemia (CLL) patients with lower T_reg_ differentiation and a shift to the T_H_1 phenotype but increased production of IFN-γ (Best et al., 2021). Recently, it was shown that neddylated Cul-4b is more abundant after T cell activation, and it is necessary to maintain the survival rate of effector CD4^+^ T cells. Since Cul-4b lacking CD4^+^ T cells are not capable of repairing DNA damage, they are more likely to undergo apoptosis (Dar et al., 2021). Taken together, neddylation is an indispensable process for T cells to function properly and survive.

For B cells, it has been reported that neddylation acts on CRLs and disrupts the NF-κB pathway in CLL B-cells. Using MLN4924 treatment, the BCL-2 homology 3-only protein (including Bim and Noxa) expression is induced in CLL cells, followed by cell apoptosis and reduced drug resistance (Godbersen et al., 2014). Alkylating agents can further promote MLN4924-induced DNA damage and apoptosis of CLL cells (Paiva et al., 2015). The effects of neddylation on B cells remain largely unknown and require further investigation.

As stated in the previous section, the inhibitor MLN4924 is a potential anti-tumor treatment, but it was found to have negative effects in treating glioblastoma (Zhou et al., 2019). Although MLN4924 can slow down tumor growth in glioblastoma, it can also elevate T-cell negative regulator programmed death-ligand 1 (PD-L1) expression by inhibiting SKP1-Cul1-F-box and WD repeat domain-containing 7 (FBXW7) activity, then lead to impaired T cell killing ability. Another study found that MLN4924 can cause impaired NEDD8-dependent clearance of misfolded proteins in dMMR/MSI tumors (deficient DNA mismatch repair/ microsatellite instability tumors), and by combination with anti-PD1, potent synergistic activity was achieved and tumor immune microenvironment was tested to be altered since the number of cytotoxic T cells and conventional CD4^+^ T cells increased whereas regulatory T cells reduced (McGrail et al., 2020).

In summary, current studies of neddylation and adaptive immunity are mostly relevant to cancer. The inhibition of neddylation can disrupt cytokines production and survival of T/B cells. MLN4924 has beneficial effects in anti-tumor therapy and autoimmune diseases, but it can also impair patients’ immune responses, making them more vulnerable to infections. Therefore, the utilization MLN4924 should be strictly monitored and studied further.

## Discussion

As a type of PTM, neddylation plays a vital role in the innate and adaptive immune responses. The neddylation process is required for immune cells to function and survive, and it is indispensable in the anti-viral pathway. And the neddylation inhibitor MLN4924 is recognized as a novel and promising cancer therapeutic strategy. However, some questions still need further research.

Firstly, although neddylation was discovered decades ago, its function and mechanism in the innate immunity and the basis of NEDD8 activation remain largely unknown. Secondly, the function of neddylation after viral infection and subsequent IFN production is debated. Thirdly, MLN4924 can suppress replication of some virus (HIV and HBV), but this treatment can also impair the anti-viral response, thus we need to learn how to balance the dual effects of MLN4924.

Once we assure these questions, the regulatory mechanisms of neddylation will be clarified and provide sound theoretical basis for the utilization of MLN4924, shedding light on treatment of cancer, viral infection and other related diseases.
